# A Two-Step Methodology to Study the Influence of Aggregation/Agglomeration of Nanoparticles on Young’s Modulus of Polymer Nanocomposites

**DOI:** 10.1186/s11671-017-2386-0

**Published:** 2017-12-15

**Authors:** Xinyue Ma, Yasser Zare, Kyong Yop Rhee

**Affiliations:** 1 Jilin Agricultural Science and Technology University, Jilin, 132101 China; 2 Young Researchers and Elites Club, Science and Research Branch, Islamic Azad University, Tehran, Iran; 3 Department of Mechanical Engineering, College of Engineering, Kyung Hee University, Yongin, 446-701 Republic of Korea; 4 Yongin, Republic of Korea

**Keywords:** Polymer nanocomposites, Aggregation/agglomeration, Multi-step method, Young’s modulus

## Abstract

A two-step technique based on micromechanical models is suggested to determine the influence of aggregated/agglomerated nanoparticles on Young’s modulus of polymer nanocomposites. The nanocomposite is assumed to include nanoparticle aggregation/agglomeration and effective matrix phases. This method is examined for different samples, and the effects of important parameters on the modulus are investigated. Moreover, the highest and the lowest levels of predicted modulus are calculated based on the current methodology. The suggested technique can correctly predict Young’s modulus for the samples assuming the aggregation/agglomeration of nanoparticles. Additionally, the aggregation/agglomeration of nanoparticles decreases Young’s modulus of polymer nanocomposites. It is demonstrated that the high modulus of nanoparticles is not sufficient to obtain a high modulus in nanocomposites, and the surface chemistry of components should be adjusted to prevent aggregation/agglomeration and to disperse nano-sized particles in the polymer matrix.

## Background

Many researchers have focused on polymer nanocomposites in recent years in order to determine the effective parameters in processing-structure-properties relationships and to optimize the overall performance as measured by mechanical, thermal, physical, and barrier properties [1–4]. A low content of nanoparticles in polymer nanocomposites produces large interfacial area, high modulus, low weight, and inexpensive products that are extremely attractive in the composite industry. Accordingly, the application of nanoparticles is an easy, efficient, and economical way to improve the performance of polymer matrices. The effects of many material and processing parameters on the properties of polymer nanocomposites containing silicate layers (nanoclay), carbon nanotubes (CNT), and inorganic fillers such as silica (SiO_2_), and calcium carbonate (CaCO_3_) have been investigated [5–8].

The size and dispersion/distribution quality of nanoparticles in polymer matrix change the general properties of polymer nanocomposites. The nanoparticles tend to aggregate and agglomerate, due to the attraction between nanoparticles such as van der Waals forces and chemical bonds [9] or the strong reduction in surface separation as filler size decreases [10]. Therefore, it is difficult to disperse the nanoparticles in polymer matrices at nanoscale. Both aggregation and agglomeration are assemblies of nanoparticles, where aggregation includes strong and dense colonies of particles, but agglomeration comprises loosely combined particles that can be disrupted by mechanical forces. Agglomeration/aggregation is evident at high filler contents, which deteriorates the nanoscale of filler and produces many defects and stress concentrations in nanocomposites [11–13]. Agglomeration/aggregation also reduces the interfacial area between polymer matrix and nanoparticles, which decreases the mechanical involvement of polymer chains in nanoparticles and eliminates the stiffening effect. Our recent findings [14, 15] and the study of Ji et al. [16] on mechanical properties have indicated that any aggregation/agglomeration severely damages the stiffening effect of nanoparticles in polymer nanocomposites.

In addition to the experimental characterization of nanocomposites, the theoretical investigations that quantify the dependence of mechanical behavior on the properties of constituent phases and the geometric morphology of nanoparticles have introduced attractive challenges in recent research. Theoretical studies may help to elucidate the experimental results and facilitate the optimal synthesis of highly promising nanocomposites. The nanoparticles in nanocomposites introduce disorder into the adjacent matrix, leading to formation of interphase zones surrounding the filler, which show different properties from bulk matrix and nanoparticles [17–19]. Theoretical studies on the interphase properties have shown attractive results, justifying the use of nanoparticles in polymer nanocomposites [20–22].

The effects of aggregation/agglomeration on the mechanical performance of nanocomposites were investigated in previous works [11, 14, 23, 24]. These studies generally considered the aggregation/agglomeration by large particles. Recently, multiscale modeling methods have been used to study the properties of nanocomposite [25–27]. In the current paper, a two-step method is suggested to examine the role of nanoparticle aggregation/agglomeration in Young’s modulus of polymer nanocomposites assuming the fraction of aggregation/agglomeration phase in nanocomposite and the portion of nanoparticles in aggregates/agglomerates. In this regard, two micromechanical models of Paul and Maxwell are applied to express Young’s modulus of nanocomposites. Numerous experimental data are presented to evaluate the predictions. Moreover, the effects of aggregation/agglomeration parameters on Young’s modulus of nanocomposites are studied.

## Methods

**Fig. 1:**
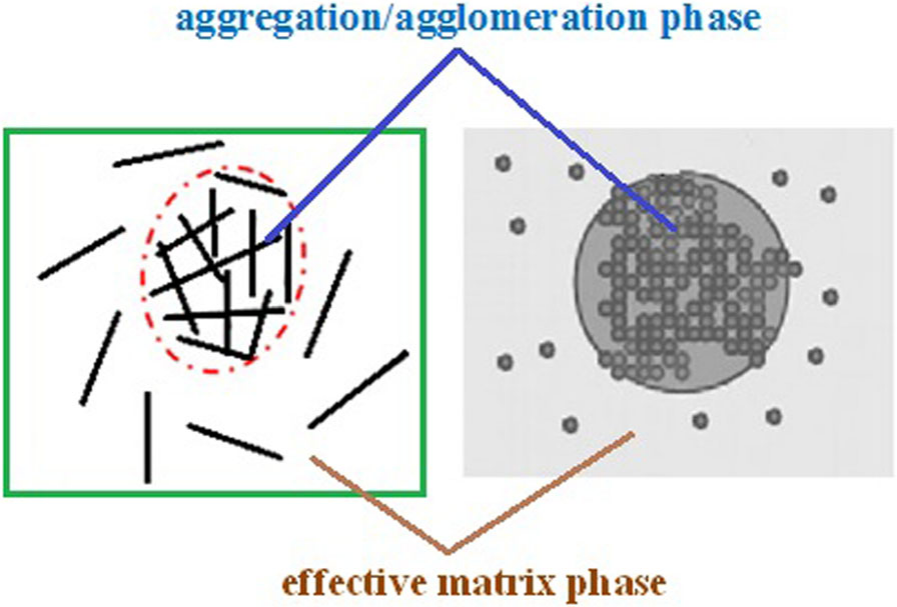
Schematic illustration of aggregation/agglomeration and effective matrix phases in polymer nanocomposites containing layered and spherical nanoparticles

When a fraction of nano-sized particles aggregates/agglomerates, a non-uniform distribution of nanoparticles is shown in nanocomposite. As a result, some nanoparticles can be assumed in spherical regions in the matrix as aggregation/agglomeration phase and others are uniformly dispersed in the polymer matrix, as illustrated in Fig. 1. Accordingly, the nanofiller shows two parts with different reinforcement which can be considered two different phases in calculation as aggregation/agglomeration and effective matrix phases which demonstrate the regions inside and outside the spheres, respectively (Figure 1).

The following two parameters are suggested for the aggregation/agglomeration level of nanoparticles in polymer nanocomposite:

1 $$ z=\frac{V_{\mathrm{agg}}}{V} $$

2 $$ y=\frac{V_f^{\mathrm{agg}}}{V_f} $$

where “*V*_agg_” and “*V*” denote the total volumes of aggregation/agglomeration phase and nanocomposite, respectively. Also, “*V*_f_^agg^” and “*V*_f_” show the volumes of nanoparticles in the aggregation/agglomeration phase and whole nanocomposite, respectively. The volume fraction of nanoparticles incorporated in the aggregation/agglomeration phase is presented by:

3 $$ {\phi}_f^{\mathrm{agg}}=\frac{V_f^{\mathrm{agg}}}{V_{\mathrm{agg}}}=\frac{y{\phi}_f}{z} $$

where “*ϕ*_*f*_” is the volume fraction of nanofiller in nanocomposites. Also, the volume fraction of well-dispersed nanoparticles incorporated in the effective matrix phase (out of aggregation/agglomeration phase) phase is calculated by:

4 $$ {\phi}_f^{\mathrm{mat}}=\frac{V_f-{V}_f^{\mathrm{agg}}}{V-{V}_{\mathrm{agg}}}=\frac{\left(1-y\right){\phi}_f}{1-z} $$

In this study, a two-step methodology based on the micromechanical models is used to determine the aggregation/agglomeration parameters (*z* and *y*) in polymer nanocomposites by Young’s modulus. Firstly, the modulus of aggregation/agglomeration and effective matrix phases is calculated by Paul’s model. Secondly, the aggregation/agglomeration phase is assumed as spherical inclusions in the effective matrix, and Young’s modulus of nanocomposite is calculated by Maxwell’s model for a composite containing dispersed particles.

Paul [28] suggested a model which assumes the macroscopically homogeneous stress in two components of composite as:

5 $$ E={E}_{\mathrm{m}}\frac{1+\left(a-1\right){\phi}_f^{2/3}}{1+\left(a-1\right)\left({\phi}_f^{2/3}-{\phi}_f\right)} $$

6 $$ a=\frac{E_{\mathrm{f}}}{E_{\mathrm{m}}} $$

where “*E*_m_” and “*E*_f_” are Young’s moduli of polymer matrix and filler phases, respectively. At the first step, the modulus of aggregation/agglomeration (*E*_agg_) and effective matrix (*E*_mat_) phases are calculated by Paul’s model through replacing “*ϕ*_*f*_” with “${\phi}_f^{agg}$” and “${\phi}_f^{mat}$” as:

7 $$ {E}_{\mathrm{agg}}={E}_{\mathrm{m}}\frac{1+\left(a-1\right){\phi_f^{\mathrm{agg}}}^{2/3}}{1+\left(a-1\right)\left({\phi_f^{\mathrm{agg}}}^{2/3}-{\phi}_f^{\mathrm{agg}}\right)}={E}_{\mathrm{m}}\frac{1+\left(a-1\right){\left(\frac{y{\phi}_f}{z}\right)}^{2/3}}{1+\left(a-1\right)\left[{\left(\frac{y{\phi}_f}{z}\right)}^{2/3}-\frac{y{\phi}_f}{z}\right]} $$

8 $$ {E}_{\mathrm{m}\mathrm{at}}={E}_{\mathrm{m}}\frac{1+\left(a-1\right){\phi_f^{\mathrm{m}\mathrm{at}}}^{2/3}}{1+\left(a-1\right)\left({\phi_f^{\mathrm{m}\mathrm{at}}}^{2/3}-{\phi}_f^{\mathrm{m}\mathrm{at}}\right)}={E}_{\mathrm{m}}\frac{1+\left(a-1\right){\left[\frac{\left(1-y\right){\phi}_f}{1-z}\right]}^{2/3}}{1+\left(a-1\right)\left[{\left(\frac{\left(1-y\right){\phi}_f}{1-z}\right)}^{2/3}-{\left(\frac{\left(1-y\right){\phi}_f}{1-z}\right)}^{2/3}\right]} $$

Also, the Maxwell model [29] for composites containing dispersed filler is given by:

9 $$ E={E}_{\mathrm{m}}\frac{1+2{\phi}_f\left(a-1\right)/\left(a+2\right)}{1-{\phi}_f\left(a-1\right)/\left(a+2\right)} $$

At the second step, the Maxwell model is applied for calculation of modulus in a composite containing an effective matrix (matrix and well-dispersed nanoparticles) and aggregation/agglomeration phases by replacing “*ϕ*_*f*_” with “*z*” (see Eq. 1), “*E*_f_” with the modulus of aggregation/agglomeration phase (*E*_agg_) and “*E*_m_” with the modulus of effective matrix (*E*_mat_) as:

10 $$ E={E}_{\mathrm{mat}}\frac{1+2z\left(k-1\right)/\left(k+2\right)}{1-z\left(k-1\right)/\left(k+2\right)} $$

11 $$ k={E}_{\mathrm{agg}}/{E}_{\mathrm{mat}} $$

which correlates Young’s modulus of nanocomposites to the moduli of aggregates/agglomerates and the effective matrix as well as the “z” parameter. When “*E*_agg_” and “*E*_mat_” from Eqs. 7 and 8 are input into the latter equations, the modulus of nanocomposites is expressed using filler concentration, filler modulus, matrix modulus, and “*z*” and “*y*” parameters. The dependency of modulus on these parameters is reasonable, because the properties of polymer and nanoparticles as well as the extent of filler aggregation/agglomeration control the modulus of nanocomposites. In the present methodology, *y* > *z* is meaningful, because ${VV}_f^{\mathrm{agg}}>{V}_f{V}_{\mathrm{agg}}$.

## Results and Discussion

**Fig. 2:**
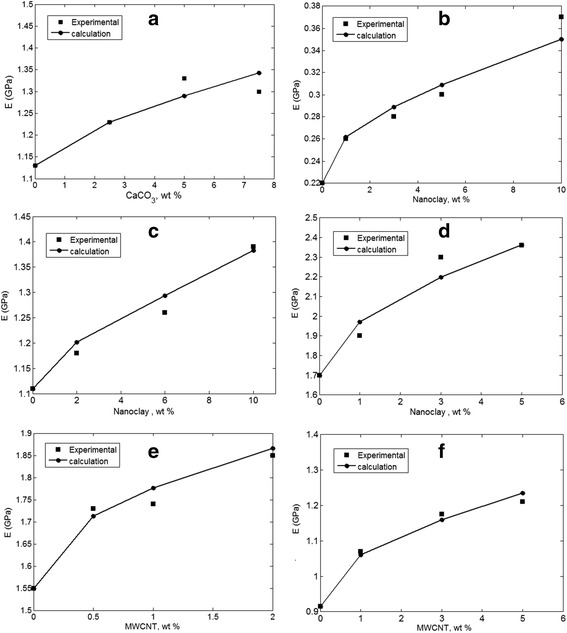
The difference between experimental and theoretical results assuming the aggregation/agglomeration of nanoparticles for **a** PVC/CaCO_3_ [30], **b** PCL/nanoclay [31], **c** ABS/nanoclay [32], **d** PLA/nanoclay [33], **e** PET/MWCNT [34], and **f** polyimide/MWCNT [35] samples

The proposed method is applied to evaluate nanoparticle aggregation/agglomeration in several samples from previous studies including PVC/CaCO_3_ [30], PCL/nanoclay [31], ABS/nanoclay [32], PLA/nanoclay [33], PET/MWCNT [34], and polyimide/MWCNT [35]. Figure 2 shows the experimental results of Young’s modulus as well as the predictions of the two-step method. The calculations properly follow the experimental data at different nanofiller concentrations, illustrating the correctness of the suggested method. However, the highest agreement between the experimental and theoretical data is obtained when the aggregation/agglomeration of nanoparticles are assumed by proper levels of “*z*” and “*y*” parameters. The highest predictions of “*z*” and “*y*” parameters are calculated as *z* = 0.2 and *y* = 0.95 for PVC/CaCO_3_ nanocomposite. Also, (*z*, *y*) values of (0.3, 0.75), (0.1, 0.99), and (0.35, 0.7) are obtained for PCL/nanoclay, PLA/nanoclay, and PET/MWCNT samples, respectively. Moreover, (*z*, *y*) levels of (0.2, 0.93) and (0.15, 0.9) are calculated for PET/MWCNT and polyimide/MWCNT nanocomposites, respectively. These levels of “*z*” and “*y*” parameters demonstrate the formation of aggregated/agglomerated nanoparticles in the mentioned nanocomposites. The small improvement of modulus in these samples confirms the weak dispersion and high level of nanoparticle accumulation in polymer matrices. For example, the addition of 7.5 wt% CaCO_3_ to PVC only increases the modulus of neat PVC (1.13 GPa) to 1.3 GPa. Also, the incorporation of 10 wt% of nanoclay in PCL only improves the modulus of neat PCL from 0.22 to 0.37 GPa. However, the nanoparticles show a high modulus compared to polymer matrices. Young’s modulus of CaCO_3_, nanoclay, and MWCNT were reported as 26, 180, and 1000 GPa [36], respectively, while Young’s modulus of the present polymer matrices hardly reaches 2.5 GPa. As a result, the aggregated/agglomerated nanoparticles significantly decrease the modulus in nanocomposites, and the present methodology suggests acceptable data for aggregation/agglomeration of nanoparticles in polymer nanocomposites.

**Fig. 3:**
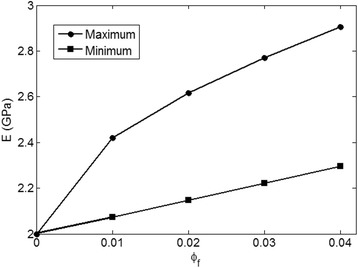
The maximum and minimum levels of modulus predicted by the present methodology at average *E*_m_ = 2 GPa and *E*_f_ = 200 GPa

The highest and smallest moduli predicted by the current methodology are calculated and illustrated in Fig. 3 at an average *E*_m_ = 2 GPa and *E*_f_ = 200 GPa. The maximum modulus is obtained by the smallest values of “*z*” and “*y*” parameters; for example, *z* = 0.00001 and *y* = 0.00001 (they cannot be 0). On the other hand, the “*y*” level of 0.99 results in the aggregation/agglomeration of all nanoparticles, which significantly reduces the modulus. Also, the highest level of “*z*” (maximum extent of agglomeration) causes the minimum modulus. “*z*” as the volume fraction of agglomerated filler in the nanocomposite is smaller than the volume fraction of all nanoparticles (*ϕ*_*f*_). So, *z* = *ϕ*_*f*_ can suggest the slightest level of modulus. The significant difference between the upper and lower values of modulus shows the important role of aggregation/agglomeration of nanoparticles in the stiffness of nanocomposites. The aggregation/agglomeration of nanoparticles in the nanocomposites greatly decreases Young’s modulus at different filler concentrations, while a fine dispersion of nanoparticles without aggregation/agglomeration produces a good modulus. Also, the high aggregation/agglomeration at great nanofiller contents decreases the rate of modulus growth upon increasing in “*ϕ*_*f*_”. Therefore, it is important to adjust the material and processing parameters to prevent the aggregation/agglomeration of nanoparticles that promote stress concentration and defects or debonding in polymer nanocomposites [37, 38].

**Fig. 4:**
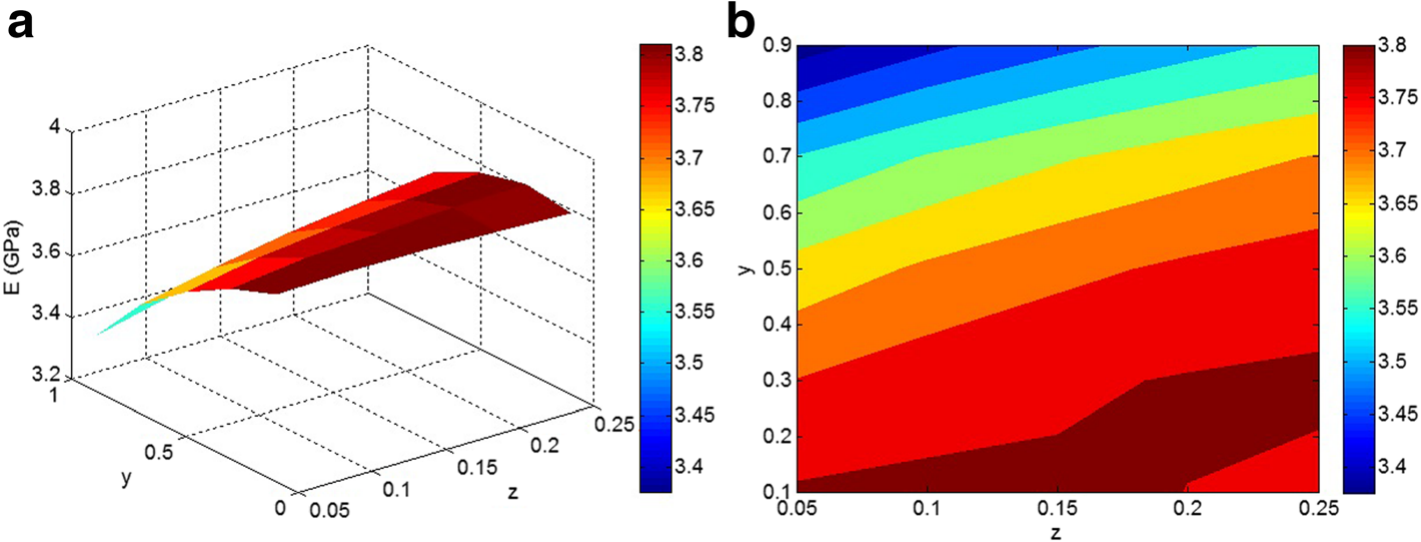
**a**, **b** The calculations of modulus by Eqs. 10–11 as a function of “*z*” and “*y*” at *E*_m_ = 3 GPa, *E*_f_ = 150 GPa, and *ϕ*_*f*_ = 0.02

Figure 4 illustrates the effects of “*z*” and “*y*” parameters on the modulus at *E*_m_ = 3 GPa, *E*_f_ = 150 GPa, and *ϕ*_*f*_= 0.02. The highest modulus is obtained at the smallest levels of “*z*” and “*y*” parameters, confirming the positive role of good dispersion/distribution of nanoparticles on the modulus of nanocomposites. However, the modulus decreases greatly as “*y*” parameter increases. According to Eq. 2, “*y*” shows the concentration of nanoparticles in the agglomeration/aggregation phase. A low modulus is observed at high “*y*” level, which shows that a large fraction of nanoparticles in the agglomeration/aggregation phase weakens a nanocomposite. Accordingly, agglomerated/aggregated nanoparticles cause a negative effect on the modulus of nanocomposites. Therefore, much effort should be made to facilitate nanoparticle dispersion/distribution in the polymer matrix, which depends on the interfacial interaction/adhesion between polymer and nanoparticles and the processing parameters. Previous studies have reported valuable results in this area and have suggested various techniques to improve this dispersion [39–41].

**Fig. 5:**
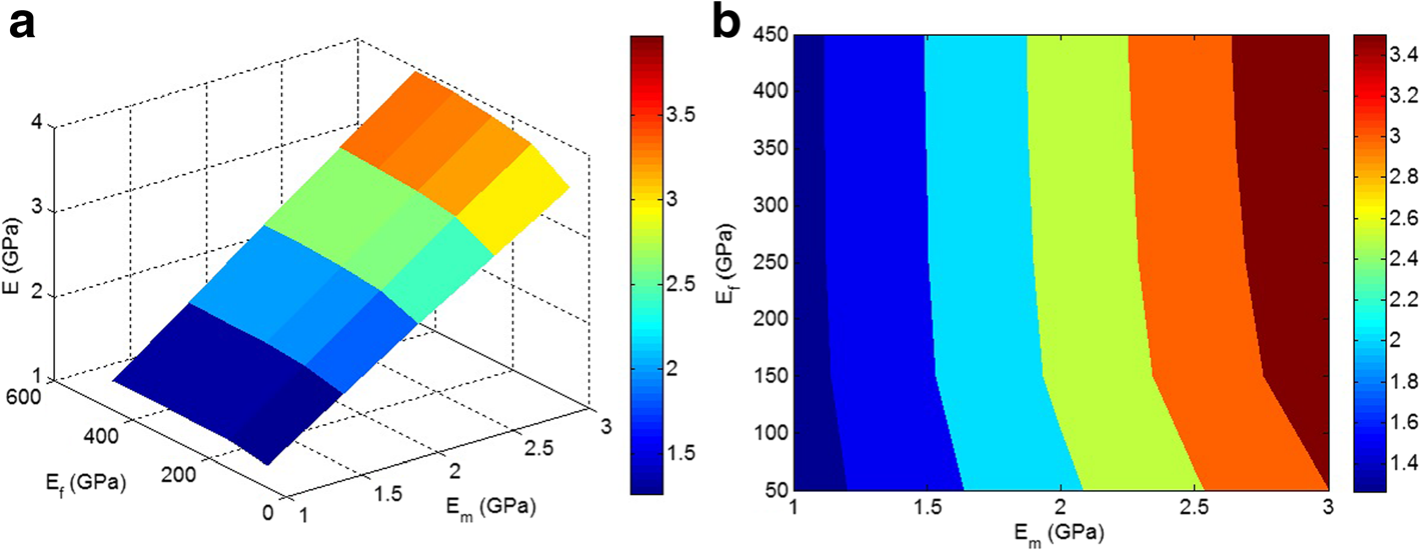
**a**, **b** The effects of “*E*_m_” and “*E*_f_” on the predicted modulus by Eqs. 10–11 at average *ϕ*_*f*_ = 0.02, *z* = 0.3, and *y* = 0.5

Figure 5 demonstrates the dependence of predicted modulus on “*E*_m_” and “*E*_f_” parameters at average *ϕ*_*f*_ = 0.02, *z* = 0.3, and *y* = 0.5 with the current technique. It is observed that the modulus depends on both “*E*_m_” and “*E*_f_” factors at low *E*_f_ < 150 GPa. However, a higher modulus of nanoparticles does not change the modulus of the nanocomposite. As a result, the modulus of nanocomposites only depends on “*E*_m_” when “*E*_f_” is higher than 150 GPa. This suggests that high nanoparticle stiffness does not play a main role in the nanocomposite modulus, and much attention should be paid to the dispersion/aggregation/agglomeration of nanoparticles.

## Conclusions

A two-step technique was suggested to determine the influences of aggregated/agglomerated nanoparticles on Young’s modulus of polymer nanocomposites. The Paul and Maxwell models were applied to calculate the moduli of aggregation/agglomeration and effective matrix phases. The predictions of the suggested methodology showed good agreement with the experimental data of different samples, assuming correct aggregation/agglomeration parameters. Accordingly, the present methodology can give acceptable results for aggregation/agglomeration of nanoparticles in polymer nanocomposites. The aggregation/agglomeration of nanoparticles significantly decreased Young’s modulus, whereas a fine dispersion of nanoparticles produced a high modulus. The highest modulus was obtained at the smallest “*z*” and “*y*” parameters, which confirmed the positive role of good dispersion/distribution of nanoparticles in the modulus of nanocomposites. However, the modulus decreases as the “*y*” parameter increased. Moreover, it was found that the excellent characteristics of nanoparticles such as high modulus are not sufficient to achieve the optimal properties in polymer nanocomposites. Accordingly, much attention should be focused on the dispersion/distribution of nanoparticles in the polymer matrix depending on the interfacial interaction/adhesion between polymer and nanoparticles and the processing parameters.
